# Interface Engineered Room‐Temperature Ferromagnetic Insulating State in Ultrathin Manganite Films

**DOI:** 10.1002/advs.201901606

**Published:** 2019-11-11

**Authors:** Weiwei Li, Bonan Zhu, Qian He, Albina Y. Borisevich, Chao Yun, Rui Wu, Ping Lu, Zhimin Qi, Qiang Wang, Aiping Chen, Haiyan Wang, Stuart A. Cavill, Kelvin H. L. Zhang, Judith L. MacManus‐Driscoll

**Affiliations:** ^1^ Department of Materials Science and Metallurgy University of Cambridge 27 Charles Babbage Road Cambridge CB3 0FS UK; ^2^ Cardiff Catalysis Institute School of Chemistry Cardiff University Main Building, Park Place Cardiff CF10 3AT UK; ^3^ Center for Nanophase Materials Sciences Oak Ridge National Laboratory Oak Ridge TN 37831 USA; ^4^ Sandia National Laboratory Albuquerque NM 87185 USA; ^5^ School of Materials Engineering Purdue University West Lafayette IN 47907 USA; ^6^ Department of Physics and Astronomy West Virginia University Morgantown WV 26506 USA; ^7^ Center for Integrated Nanotechnologies Los Alamos National Laboratory Los Alamos NM 87545 USA; ^8^ Department of Physics University of York York YO10 5DD UK; ^9^ Diamond Light Source Didcot OX11 0DE UK; ^10^ State Key Laboratory of Physical Chemistry of Solid Surfaces College of Chemistry and Chemical Engineering Xiamen University Xiamen 361005 China

**Keywords:** ABO_3_ perovskite oxides, ferromagnetic insulators, interface engineering, manganite thin films, octahedral proximity effect

## Abstract

Ultrathin epitaxial films of ferromagnetic insulators (FMIs) with Curie temperatures near room temperature are critically needed for use in dissipationless quantum computation and spintronic devices. However, such materials are extremely rare. Here, a room‐temperature FMI is achieved in ultrathin La_0.9_Ba_0.1_MnO_3_ films grown on SrTiO_3_ substrates via an interface proximity effect. Detailed scanning transmission electron microscopy images clearly demonstrate that MnO_6_ octahedral rotations in La_0.9_Ba_0.1_MnO_3_ close to the interface are strongly suppressed. As determined from in situ X‐ray photoemission spectroscopy, O *K*‐edge X‐ray absorption spectroscopy, and density functional theory, the realization of the FMI state arises from a reduction of Mn e_g_ bandwidth caused by the quenched MnO_6_ octahedral rotations. The emerging FMI state in La_0.9_Ba_0.1_MnO_3_ together with necessary coherent interface achieved with the perovskite substrate gives very high potential for future high performance electronic devices.

## Graphical abstract

Ultrathin ferromagnetic insulators with Curie temperatures above room temperature are critically needed for developing dissipationless quantum electronic and spintronic devices. Unfortunately, such materials are extremely rare in nature. Room‐temperature ferromagnetic insulating states are successfully synthesized by an interfacial octahedral proximity effect in ultrathin La_0.9_Ba_0.1_MnO_3_ films, which could serve as promising candidates for future oxide‐based electronic devices.

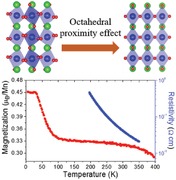

Transition metal ABO_3_ perovskite oxides are promising materials for future technologies as their physical properties can be readily tailored via strain engineering,^1^, ^2^, ^3^ interface engineering (e.g., polar discontinuity^4^ and charge transfer^5^), and defect engineering,^6^, ^7^, ^8^, ^9^, ^10^, ^11^ thus allowing one to design new materials with novel properties. Note that, in ABO_3_ perovskites, spin, charge, and orbital orders are intimately correlated with the corner‐connected BO_6_ octahedra.^12^, ^13^ To retain the corner connectivity of oxygen octahedra across an interface, an interfacial octahedral proximity effect emerges that can imprint the rotation of the BO_6_ octahedra in one perovskite onto the adjacent perovskite.^13^, ^14^, ^15^ Also, the B—O bonds determine the orbital order and exchange interactions, the interfacial octahedral proximity effect at the heterointerface has started to be explored for engineering novel functionalities, providing a new route for tuning the electronic and magnetic properties of perovskites at atomic scale.^16^, ^17^, ^18^, ^19^, ^20^, ^21^, ^22^, ^23^, ^24^, ^25^, ^26^, ^27^

Ultrathin ferromagnetic insulators (FMIs, 1–4 nm) with Curie temperatures above room temperature and forming coherent interface with electrodes are strongly needed for generating pure spin‐polarized currents in next‐generation dissipationless quantum electronic and spintronic devices.^28^, ^29^, ^30^, ^31^, ^32^ Unfortunately, current candidate ferromagnetic insulating materials have an extremely low *T*_C_ such as EuS (*T*_C_ ≈ 16.6 K),^33^, ^34^ EuO (*T*_C_ ≈ 69.3 K),^33^, ^34^ BiMnO_3_ (*T*_C_ ≈ 105 K),^34^, ^35^ La_0.1_Bi_0.9_MnO_3_ (*T*_C_ ≈ 90 K),^31^ and LaCoO_3_ (*T*_C_ ≈ 85 K).^36^ Their low spin‐filter efficiency, low *T*_C_, and low exchange splitting, due to the poor quality in ultrathin film form, pose significant limitations for applications in devices. Some spinel ferrimagnetic insulators such as NiFe_2_O_4_ (*T*_C_ ≈ 850 K),^34^, ^37^ CoFe_2_O_4_ (*T*_C_ ≈ 796 K),^34^, ^38^ and double‐perovskite ferromagnetic semiconductor La_2_NiMnO_6_ (*T*_C_ ≈ 280 K)^39^ have *T*_C_ above or very close to room temperature. However, their complex structure, tendency to form antisite defects, and poor chemical compatibility are obstacles to achieving high‐quality interfaces with electrodes, which significantly reduce the performance of devices. Consequently, ferromagnetic insulators with a higher *T*_C_ need to be further explored for realizing the spin‐polarization at room temperature.

Room‐temperature ferromagnetic metallic manganite perovskites are excellent electrode materials and are widely used for the spin detection layer.^31^ To realize high tunneling magnetoresistive devices, a similar‐composition, isostructural ferromagnetic manganite insulating layer between the electrodes is actually needed. Such a similar‐composition and isostructural layer will enable perfect interfaces to be formed, thus preventing the degradation of interface spin transfer.

In this study, we explore the utilization of the interfacial octahedral proximity effect to achieve a room‐temperature ferromagnetic insulating state in ultrathin La_0.9_Ba_0.1_MnO_3_ (LBMO) films grown on SrTiO_3_ (STO) (001) substrates. Bulk LBMO (pseudocubic lattice parameter ≈3.89 Å) is a ferromagnetic insulator (*T*_C_ ≈ 185 K) whereas thin films of LBMO grown on STO substrates typically show a ferromagnetic metallic state with *T*_C_ of 250–295 K.^3^ The modified properties were attributed to in‐plane tensile strain from STO substrate. Here, the interfacial octahedral proximity effect on the electronic structures and ferromagnetic insulating properties of LBMO films is studied in detail. From both experiments (scanning transmission electron microscopy (STEM), X‐ray magnetic circular dichroism (XMCD) and linear dichroism (XLD), in situ X‐ray photoemission spectroscopy (XPS), and O *K*‐edge X‐ray absorption spectroscopy (XAS)) and density functional theory (DFT) calculations, we identified that, when MnO_6_ octahedral rotations in LBMO close to the interface are strongly suppressed, the Mn e_g_ bandwidth is reduced and magnetic interactions are enhanced simultaneously. This leads to the emergence of ferromagnetic insulating state in an ultrathin (5 unit cell) LBMO film with a remarkable *T*_C_ above room temperature.

We used pulsed laser deposition to fabricate high‐quality LBMO epitaxial thin films on TiO_2_‐terminated STO (001) substrates. The film thickness was controlled by in situ monitoring of the reflection high‐energy electron diffraction (RHEED) oscillations.^40^ Structural characterizations (Figure S1, Supporting Information) show LBMO films with atomically smooth surface, high crystalline quality, and fully strained to STO substrate. As shown in **Figure** **1**a, at room temperature, orthorhombic LBMO (bulk) exhibits *a* ^−^ *a* ^−^ *c* ^+^ rotation pattern (*Pbnm*) with Mn–O–Mn angles of 159.20°(in‐plane) and 162.18°(out‐of‐plane),^41^, ^42^ while cubic STO crystallizes with *a* ^0^ *a* ^0^ *a* ^0^ pattern (*Pm‐3m*).^42^ It is expected that this symmetry difference will introduce an interfacial octahedral coupling region at the LBMO/STO interface. In other words, the oxygen atoms need to realign at the interface for retaining corner connectivity of oxygen octahedra.

**Figure 1:**
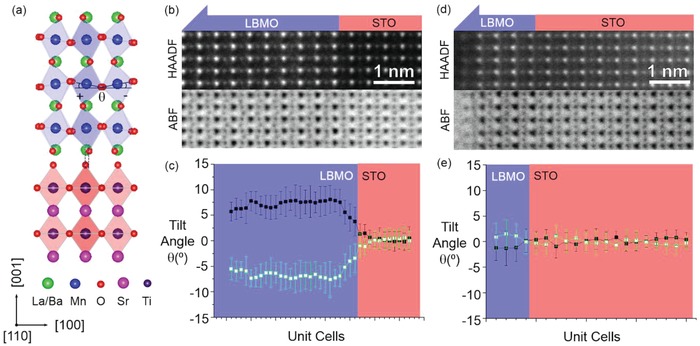
a) Schematic models of oxygen octahedral patterns for bulk LBMO (top panel) and STO (bottom panel), viewed from the pseudocubic [110] direction. HAADF and ABF‐STEM images of b) 40 uc and d) 5 uc LBMO, viewed from the pseudocubic [110] direction. Plane‐averaged octahedral tilt angle of c) 40 uc and e) 5 uc LBMO.

For STEM images of ABO_3_ perovskites, the relatively heavier cation sublattices can be directly visualized in the high angle annular dark field (HAADF) mode, while oxygen sublattices can be observed using annular bright‐field (ABF) mode. Therefore, BO_6_ octahedral tilt angle and full 3D rotation pattern can be determined using the combination of STEM images taken in HAADF and ABF modes.^43^ Figure 1b shows HAADF and ABF STEM images of the 40 uc LBMO film grown on STO, viewed from pseudocubic [110] direction. The observed structure is consistent with two out‐of‐phase (–, –) rotation directions in the in‐plane. The plane averaged and projected tilt angle is determined from the ABF STEM image and is shown in Figure 1c. While the film away from the LBMO/STO interface has a projected tilt angle close to the value of bulk LBMO (≈8°),^41^ there is a clear interfacial octahedral coupling region of ≈5–6 uc in the LBMO close to the LBMO/STO interface, where the tilt angle is constrained by the STO substrate. In contrast, a very different structure was observed when the thickness of the LBMO film is reduced to 5 uc. As shown in Figure 1d, from the HAADF and ABF STEM images along the pseudocubic [110] direction, no projected MnO_6_ tilting was observed. The plane averaged and projected tilt angle determined from the ABF STEM image is close to 0° (Figure 1e). In order to exclude the possibility of having mixed phase (+,‐) tilt along the in‐plane direction, additional observation was made along the pseudocubic [100] direction (Figure S2, Supporting Information), and again no tilt was found. From these observations, we can safely conclude that the MnO_6_ octahedral rotations in the ultrathin LBMO (i.e., 5 uc) have been quenched. Combined with octahedral rotations observed in the 40 uc film, it can be envisaged that, with decreasing the film thickness, the MnO_6_ octahedral rotations are significantly modified by the interfacial octahedral proximity effect.

Temperature dependent magnetization and resistance measurements are presented in **Figure** **2**a,b and Figure S3 in the Supporting Information. The 40 uc film exhibits a metal‐to‐insulator transition and a *T*_C_ of around 291 K (Figure 2a), in agreement with previous work.^3^ However, as shown in Figure 2b, the 5 uc film shows different behaviors. With increasing temperature, the magnetization curve shows two transition temperatures with *T*_C1_ ≈ 100 K and *T*_C2_ > 400 K with a sizable magnetization of 0.29 *µ*_B_/Mn retained at 400 K. Meanwhile, the resistance curve shows an insulating behavior, indicating the 5 uc film with magnetic insulating properties. To confirm the observed magnetic results, we performed XMCD studies in a grazing incidence geometry and total electron yield (TEY) detection on the 5 uc and 40 uc films, as shown in Figure 2c,d. It has been demonstrated that XMCD enables the detection of subtle (≈0.005 *µ*_B_/atom) and element‐specific magnetic moment, thus excluding the contribution from magnetic impurities.^44^ The XMCD spectra of the Mn *L*_2,3_ edges measured at 2 K under 4 T are shown in Figure 2c. The line shapes of spectra are similar to the reported data for LaMnO_3_ and La_0.7_Sr_0.3_MnO_3_.^45^, ^46^ XMCD spectra clearly reveal strong dichroism, indicating a net ferromagnetic moment associated with Mn cations in the films. The large XMCD signal in the 5 uc film is comparable to that observed in LaMnO_3_ with a thickness of 9 uc and hole‐doped La_0.7_Sr_0.3_MnO_3_ with a thickness of 40 nm.^45^, ^47^ Mn XMCD of the 5 uc film at 2 K confirms the first transition temperature (*T*_C1_ ≈ 100 K; Figure 2b) derives from the ferromagnetic transition. To further investigate the second transition temperature (*T*_C2_ > 400 K; Figure 2b), XMCD measurements at the Mn *L*_2,3_ edges under 0 T were carried out and XMCD spectra are displayed in Figure 2d. As expected, zero XMCD is observed in the 40 uc films at 300 K which agrees well with the ferromagnetic *T*_C_ (≈291 K; Figure 2a) below 300 K. Due to the high sensitivity of the XMCD measurements, small magnetic moments can also be detected and for the 5 uc film, a nonzero XMCD is clearly detected at 300 K indicating the 5 uc film is ferromagnetic at room temperature. Together with transport data, our detailed magnetic measurements confirm that room‐temperature ferromagnetic insulating state is achieved in the 5 uc LBMO film.

**Figure 2:**
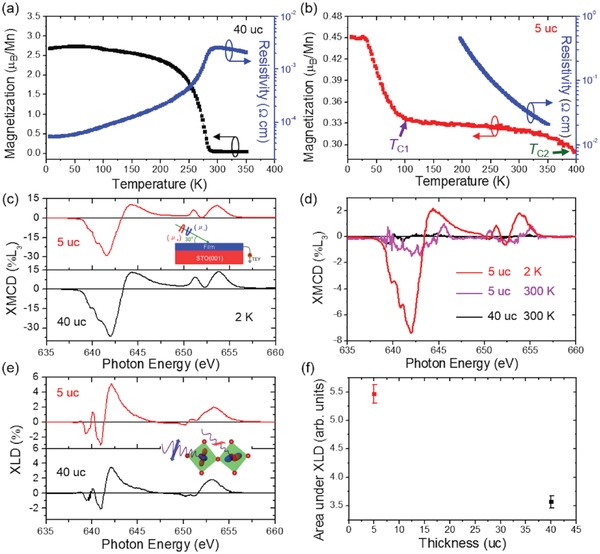
Temperature dependent magnetization and resistivity of a) 40 uc and b) 5 uc LBMO measured by 100 Oe field along the in‐plane direction. c) XMCD under 4 T, d) XMCD under 0 T, and e) XLD measurements of 5 uc (red and magenta) and 40 uc (black) LBMO. f) Integrated area of XLD signal calculated from 648–660 eV as a function of thickness.

We found that the Ti close to the LBMO/STO interface in the 5 uc film exhibits a mixed 3+/4+ oxidation state, indicating the charge transfer also occur at the interface similar to the LaAlO_3_/SrTiO_3_ and La_0.7_Sr_0.3_MnO_3_/SrTiO_3_ interfaces.^44^, ^48^, ^49^ It is also noted that XAS spectra at Mn *L*_2,3_ edges reveal no variation with film thickness (Figure S4, Supporting Information), suggesting the La_0.9_Ba_0.1_O layer donates electrons to the interfacial Ti. Furthermore, by applying sum rules for Ti and Mn *L*_2,3_ edges,^50^ the Mn–O–Ti superexchange interaction is determined to be ferromagnetic at the interface (Figure S5, Supporting Information). In particular, it is noteworthy that the Ti magnetic moment is strongly coupled parallel to the Mn magnetic moment till the first ferromagnetic transition temperature (*T*_C1_ ≈ 100 K; Figure S6, Supporting Information).

To determine the orbital occupancy, XLD measurements at the Mn *L*_2,3_ edges were performed at 300 K without a magnetic field in grazing incidence geometry. The XLD spectra provide information on the empty Mn 3d orbital states. Specifically, the smaller (larger) absorption for in‐plane polarization suggests more out‐of‐plane (in‐plane) empty states in the e_g_ band and thus a higher occupancy in the in‐plane (out‐of‐plane) orbitals.^51^, ^52^ As shown in Figure 2e, similar XLD spectra are observed in the 5 uc and 40 uc films and the dichroic signals around the high‐energy *L*_2_ absorption peak display a positive sign, implying a preferential occupation of the out‐of‐plane d_3_ *_z_* ^2^_−_ *_r_* ^2^ orbitals.^52^ The unexpected out‐of‐plane d_3_ *_z_* ^2^_−_ *_r_* ^2^ occupancy in e_g_ orbital shown in the 40 uc film under tensile strain can be explained by a vacuum‐interface induced extra contribution of the orbital configuration, favoring occupancy of the out‐of‐plane d_3_ *_z_* ^2^_−_ *_r_* ^2^ states. This has been demonstrated in La_0.67_Sr_0.33_MnO_3_ films under tensile strain.^52^ To quantify the orbital occupancy, the area under the *L*_2_ absorption peak (648–660 eV) in the XLD spectra was calculated by integration, as presented in Figure 2f. The data reveals that the d_3_ *_z_* ^2^_−_ *_r_* ^2^ orbital occupancy increases with a decrease of the film thickness, in agreement with an enhanced occupation of d_3_ *_z_* ^2^_−_ *_r_* ^2^ orbital in the La_0.67_Sr_0.33_MnO_3_ films caused by surface effect.^52^ However, based on the interfacial Mn–O–Ti ferromagnetic interaction and Mn 3d orbital reconstruction, we are not able to elucidate the origin and nature of the second transition temperature (*T*_C2_) observed in the 5 uc film.

To further elucidate the origin of room‐temperature ferromagnetic insulating state in the 5 uc films, we performed detailed investigations on the electronic structures. **Figure** **3**a shows in situ XPS valence band (VB) spectra for the 5 uc and 40 uc films. Compared with the spin‐resolved photoemission spectroscopy of La_0.7_Sr_0.3_MnO_3_ films,^46^ it can be seen that the Mn 3d states split into the majority spin t_2g_ (≈2.4 eV) and e_g_ (≈1.05 eV) states at the top of VB. The VB maximum (VBM) for the 5 uc film is located at 0.42 eV higher binding energy than that for the 40 uc film. Also, no visible intensity at the Fermi level (*E*_f_) is observed for the 5 uc film (Figure S7, Supporting Information). These results further confirm the films change from a metallic state to an insulating state with decreasing film thickness, consistent with the transport measurement.

**Figure 3:**
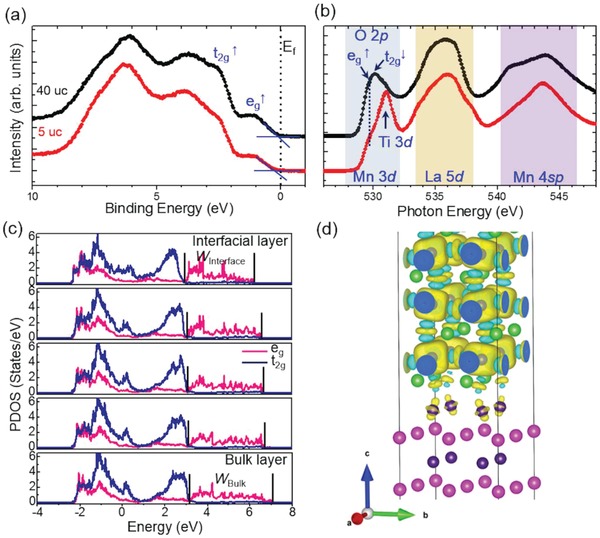
a) In situ XPS valence band and b) O *K*‐edge XAS spectra for the 5 uc and 40 uc LBMO films. c) Partial density of states spectra of majority spin Mn e_g_ and t_2g_ orbitals. d) An isosurface plot of the spin density at the STO–LBMO interface. Positive and negative isosurface are labeled with yellow and blue colors.

O *K*‐edge XAS spectra are shown in Figure 3b. The O *K*‐edge XAS probes the transition from O 1s to the unoccupied states derived mainly from the hybridization of O 2p states with Mn 3d states, La 5d states, and Mn 4sp states. The spectrum can be qualitatively related to the unoccupied density of states above *E*_f_.^53^ The overall line shape of the 5 uc and 40 uc films agrees well with the reported spectra of La_1−_ *_x_*Sr*_x_*MnO_3_,^54^, ^55^, ^56^ apart from an additional feature at ≈530.9 eV associated with Ti 3d states from the STO substrate.^57^ The XLD spectra determine the e_g_ band with the occupancy of the out‐of‐plane d_3_ *_z_* ^2^_−_ *_r_* ^2^ states, which can be assigned to be the low‐energy e_g_^↑^orbital. Consequently, in the O *K*‐edge XAS spectra, the first peak feature just below 530 eV is attributed to an accidental superposition of majority e_g_^↑^ with d*_x_* ^2^_−_ *_y_* ^2^ symmetry and minority t_2g_^↓^bands. The first peak feature shares the same peak position in the 5 uc and 40 uc films, suggesting no change of the oxidation state of the Mn atoms. However, comparing with the 40 uc film, the pre‐edge position of the first peak in the 5 uc film moves toward higher photon energies (Figure S8, Supporting Information). Combined with the VBM shift toward higher binding energies in the 5 uc film, it can be concluded that the e_g_ bandwidth is reduced and a bandgap emerges with decreasing film thickness.^58^ Note that, in bulk manganites, the e_g_ bandwidth increases as the Mn—O—Mn bond angles increase and the Mn—O bond lengths decrease. The reduction of the e_g_ bandwidth in the 5 uc film can be explained by the increase of both of the Mn—O—Mn bond angles and Mn—O bond lengths caused by the tensile strain and the suppressed MnO_6_ octahedral rotations.

To gain further insight, we performed first‐principles DFT calculations of the LBMO/STO interface. DFT calculations confirm that the MnO_6_ octahedral rotations are suppressed at the interface (Figure S9a, Supporting Information), consistent with suppression of interfacial MnO_6_ octahedral rotations observed in the 5 uc and 40 uc films (Figure 1b–e). Also, a reduction of Mn e_g_ bandwidth from 3.85 eV in the bulk layer to 3.30 eV in the interfacial layer is clearly observed (Figure 3c). The d_3_ *_z_* ^2^_−_ *_r_* ^2^ orbital occupancy of e_g_ band is also supported by the DFT calculations through partial density of states analysis. Additionally, the very first layer of Ti at the LBMO/STO interface is spin‐polarized, indicating the charge is transferred from LBMO to STO (Figure 3d). It can be seen that the Ti spin polarization has *d*_3_ *_z_* ^2^_−_ *_r_* ^2^ orbital shape. Moreover, the interfacial Mn–O–Ti magnetic interaction is ferromagnetic (Figure 3d), in good agreement with the experimental results (Figure S5, Supporting Information).

Based on the above discussion, a phase diagram is constructed in **Figure** **4** to illustrate the overall temperature and thickness dependent magnetization and conductivity. Recent experiments in La_0.7_Sr_0.3_MnO_3_ and Ca_0.5_Sr_0.5_TiO_3_ have established that the octahedral proximity effect only gives a modulation of the octahedral tilt up to ≈8 uc.^22^, ^23^ It has also been demonstrated that magnetic interactions and magnetic ordering temperature can be enhanced in La_0.5_Sr_0.5_MnO_3_,^25^ La_0.67_Sr_0.33_MnO_3_,^59^ and SrIrO_3_ 60 via the suppressed MnO_6_ and IrO_6_ octahedral rotations, respectively. In current study, the MnO_6_ octahedral rotations in the 5 uc film are quenched due to octahedral proximity effect (Figure 1c). Consequently, the Mn—O—Mn bond angles are significantly increased and then the magnetic exchange interaction within LBMO can be drastically enhanced. As a consequence, a second magnetic transition temperature (*T*_C2_) emerges in the 5 uc film (Figure 2b). When the temperature is decreased, the interfacial Mn–O–Ti and Mn–O–Mn magnetic interactions dominate giving rise to the first ferromagnetic temperature (*T*_C1_; Figure 2b; Figure S6d, Supporting Information). Similar to the induced insulating state in 3 uc SrIrO_3_ film by the suppression of IrO_6_ octahedral rotations,^60^ the quenched MnO_6_ octahedral rotations in 5 uc film also reduce the e_g_ bandwidth and consequently induce an insulating state. However, for the thicker LBMO films, the interfacial octahedral proximity effect decays quickly and the double exchange interaction starts to control the magnetic and transport behaviors. It should also be noted that the ferromagnetic metallic to ferromagnetic insulating transition happens at the critical thickness of 7–8 uc, which coincides with the length scale of octahedral proximity effect. These results demonstrate that the octahedral proximity effect is responsible for the emerged room‐temperature ferromagnetic insulating phase in the 5 uc LBMO film.

**Figure 4:**
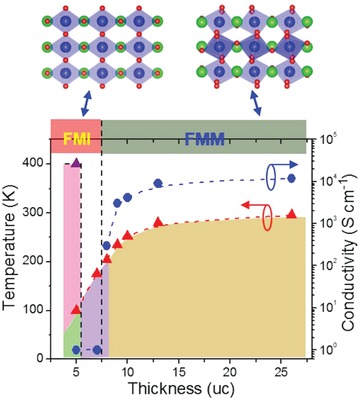
Top panel: schematic models of oxygen octahedral patterns for LBMO with quenched (left) and normal (right) MnO_6_ octahedral rotations. Bottom panel: thickness dependent magnetic transition temperature and the residual conductivity at 5 K.

By engineering the interfacial octahedral proximity effect, from both experiment and theory, we achieved a ferromagnetic insulating state in the ultrathin LBMO films up to 300 K. Through a suppression of MnO_6_ octahedral rotations, the magnetic exchange interactions are enhanced, which increases the magnetic ordering temperature. Furthermore, the Mn e_g_ bandwidth is reduced, which produces an insulating state. Ultimately, an FMI state is realized in LBMO films of <8 uc thickness and the ferromagnetic *T*_C_ is above room temperature in films of 5 uc thickness. Not only are the novel octahedral proximity‐engineered ultrathin FMI films obtained here of direct importance for the development of novel oxide quantum materials for energy efficiency quantum electronic and spintronic devices, but the results also lend themselves to the use of the octahedral proximity effect in perovskite oxide superlattice or multilayer heterostructures, where the presence of multiple interfaces would further amplify the interfacial proximity effect for engineering novel functionalities.

## Experimental Section

*Film Fabrication and Basic Characterization*: Details on sample preparations can be found elsewhere.^40^ The crystalline nature of the films was investigated by X‐ray diffraction (XRD) on a high‐resolution X‐ray diffractometer (Empyrean, PANalytical, The Netherlands) using Cu Kα radiation (λ = 1.5405 Å). The films' surface morphologies were examined by atomic force microscopy (AFM).

*Transmission Electron Microscopy*: Cross‐sectional specimen oriented along [100]_pc_ and [110]_pc_ directions for STEM analysis were prepared by conventional mechanical thinning, precision polishing, and ion milling. HAADF and ABF STEM images were taken using Nion UltraSTEM operating at 200 kV, equipped with a cold field‐emission electron gun and a corrector of third‐ and fifth‐order aberrations. The convergence semiangle for the electron probe was about 30 mrad. HAADF signals for the samples were collected from a detector angle range with an inner collection angle of ≈63 mrad. Thirty quickly scanned images (0.5 µs per pixel, with pixel size of 5–10 pm) were aligned via autocorrelation and displayed as sum image, which were wiener filtered to reduce noise. Image analysis was done via Digital Micrograph and ImageJ scripts.

*Magnetic and Transport Characterization*: The magnetization measurements for LBMO films were performed with a Quantum Design MPMS3 SQUID‐VSM magnetometer. Resistance was measured by a Quantum Design Physical Property Measurement System (PPMS).

*X‐Ray Photoemission Spectroscopy*: In situ X‐ray photoemission spectroscopy was undertaken by a monochromatic Al Kα X‐ray source (*hν* = 1486.6 eV) using a SPECS PHOIBOS 150 electron energy analyzer with a total energy resolution of 0.5 eV. The measurements were performed at 300 K. The Fermi level of the films was calibrated using a polycrystalline Au foil.

*X‐Ray Absorption Spectroscopy*: X‐ray absorption spectroscopy measurements with polarization dependence were performed at the I06 beamline of the Diamond Light Source. The XMCD measurements were carried out using a TEY detection in grazing incidence geometry. XMCD measurements were performed between 2 and 300 K in a 4 T magnetic field applied in the *a*–*b* plane of the films, parallel to the beam propagation direction. To ensure that the XMCD signal is of magnetic origin, the magnetic field was applied in the opposite direction to verify the sign of the XMCD reversed. XLD measurements were carried out at 300 K without a magnetic field and a TEY detection was used in grazing incidence geometry. The XLD spectra were obtained by the intensity difference (*I*_v_ − *I*_h_) between the spectra measured with horizontal (*E*_h_) and vertical (*E*_v_) linear polarizations. Due to the second transition temperature observed in the 5 uc films above 300 K, the XLD spectra might be slightly affected by the magnetic contribution.

*Density Functional Theory Calculations*: DFT calculations were carried out using the plane wave pseudopotential code CASTEP.^61^ The Perdew–Burke–Ernzerhof (PBE) exchange–correlation functional was used.^62^ A plane wave cut off energy of 700 eV and a 3 × 3 × 1 k‐point grid were used. The valence state 2s and 2p of O, 3s, 3p, 3d, and 4s of Ti, 3s, 3p, 3d, and 4s of Mn, 4s, 4p, and 5s of Sr, and 5s, 5p, and 6p of Ba were treated with on‐the‐fly generated core‐corrected ultrasoft pseudopotentials. Density of states were calculated using OptaDOS with adaptive broadening.^63^, ^64^ A slab configuration was used to model the LBMO thin film on the STO substrate. The simulation cell consisted of 2 × 2 × 4 unit cells of STO and 2 × 2 × 8 pseudocubic cells of LBMO, as shown in Figure S9a in the Supporting Information. A vacuum gap of 15 Å was included to minimize interactions between the slab and its periodic images. Doping was introduced explicitly by replacing one in eight La atoms by Ba. To improve the inadequate description of the correlated d electrons in standard DFT calculations, the Hubbard *U* correction was used with *U* = 3 eV for the Mn 3d electrons.^65^ The structure was optimized with a fixed unit cell. The STO substrate was chosen to be fully fixed to maximize the pinning of the octahedron network caused by the substrate, since the strong pinning was observed experimentally. The in‐plane lattice parameters of the bilayer structure were fixed to 3.98 Å to be consistent with the experimental tensile strain state of LBMO. It is well known that PBE functional systematically overestimates the lattice constants of solids. Calculations were also performed using the PBE relaxed lattice constant of the STO substrate, 3.937 Å, as in‐plane lattice constant and similar results were obtained. The AiiDA framework was used to manage the calculations and preserve their provenance.^66^

## Conflict of Interest

The authors declare no conflict of interest.

## Supporting information

Supporting Information
