# Supplementary material for: Interface Engineered Room‐Temperature Ferromagnetic Insulating State in Ultrathin Manganite Films
Source: Adv Sci (Weinh). 2019 Nov 11;7(1):1901606. doi: 10.1002/advs.201901606 (PMC6947487; doi:10.1002/advs.201901606)
Supplement: Supplementary file 1 — Supporting Information [file ADVS-7-1901606-s001.pdf]

Copyright WILEY-VCH Verlag GmbH & Co. KGaA, 69469 Weinheim, Germany, 2019.

ADVANCED SCIENCE
Open Access

Supporting Information

for *Adv. Sci.,* DOI: 10.1002/advs.201901606

Interface Engineered Room-Temperature Ferromagnetic Insulating State in Ultrathin Manganite Films

Weiwei Li, Bonan Zhu, Qian He, Albina Y. Borisevich, Chao Yun, Rui Wu, Ping Lu, Zhimin Qi, Qiang Wang, Aiping Chen, Haiyan Wang, Stuart A. Cavill, Kelvin H. L. Zhang,* and Judith L. MacManus-Driscoll**

Copyright WILEY-VCH Verlag GmbH & Co. KGaA, 69469 Weinheim, Germany, 2019.

Supporting Information

Interface Engineered Room-Temperature Ferromagnetic Insulating State in Ultrathin Manganite Films

Weiwei Li[†]*, *Bonan Zhu*[†], *Qian He*[†], *Albina Y. Borisevich, Chao Yun, Rui Wu, Ping Lu, Zhimin Qi, Qiang Wang, Aiping Chen, Haiyan Wang, Stuart A. Cavill, Kelvin H. L. Zhang*, and Judith L. MacManus-Driscoll**

[†]*W.L., B.Z., and Q. H. contributed equally to this work.*

*Email: wl337@cam.ac.uk, kelvinzhang@xmu.edu.cn, jld35@cam.ac.uk

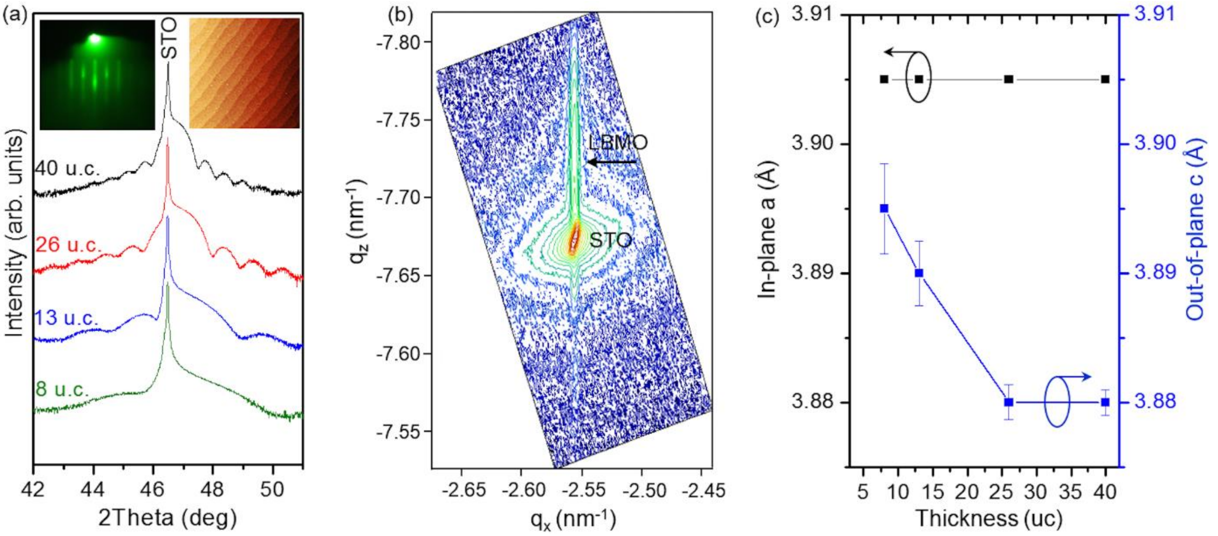

Figure S1. (a) X-ray diffraction θ-2θ detailed scan around (002) peak. The insets are the reflection high-energy electron diffraction (RHEED) pattern (top left) and 5 × 5 *u*m AFM morphology image (top right) for the 40 *uc* LBMO. (b) Reciprocal space map of (103) Bragg reflection of LBMO (40 *uc*)/STO. (c) In-plane and out-of-plane lattice constant as a function of thickness.

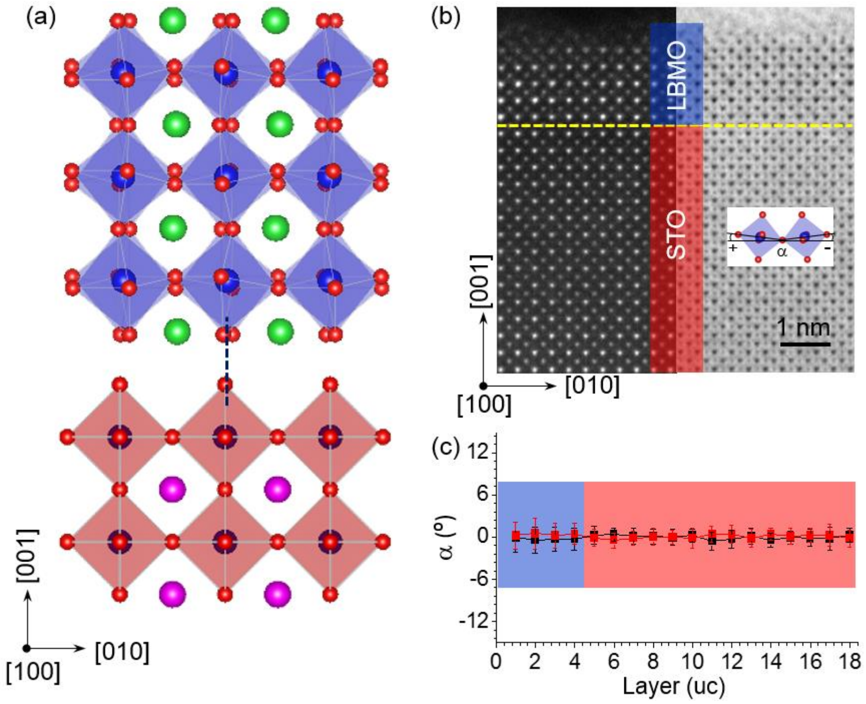

Figure S2. (a) Schematic models of oxygen octahedral patterns for LBMO (top panel) and STO (bottom panel), viewed from the pseudo cubic [100] direction. (b) LBMO 5 *uc* grown on STO substrate: Left is a high-magnification STEM HAADF image and right is STEM ABF image, viewed from the pseudo cubic [100] direction. (c) Plane-averaged octahedral title angle.

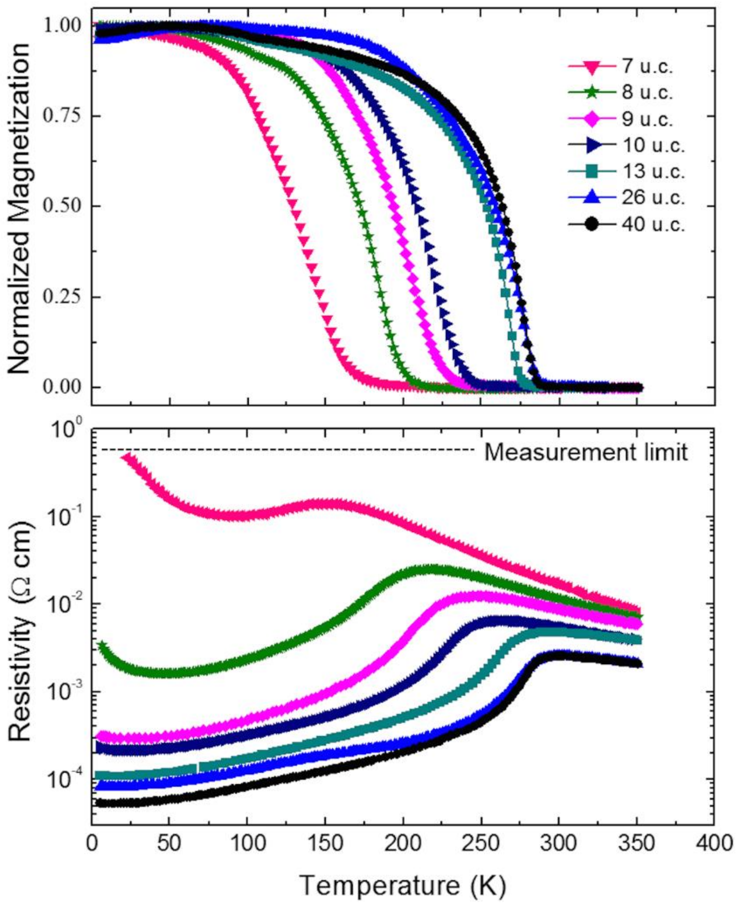

Figure S3. Temperature dependent magnetization (top panel) and resistivity (bottom panel) of LBMO with different thickness.

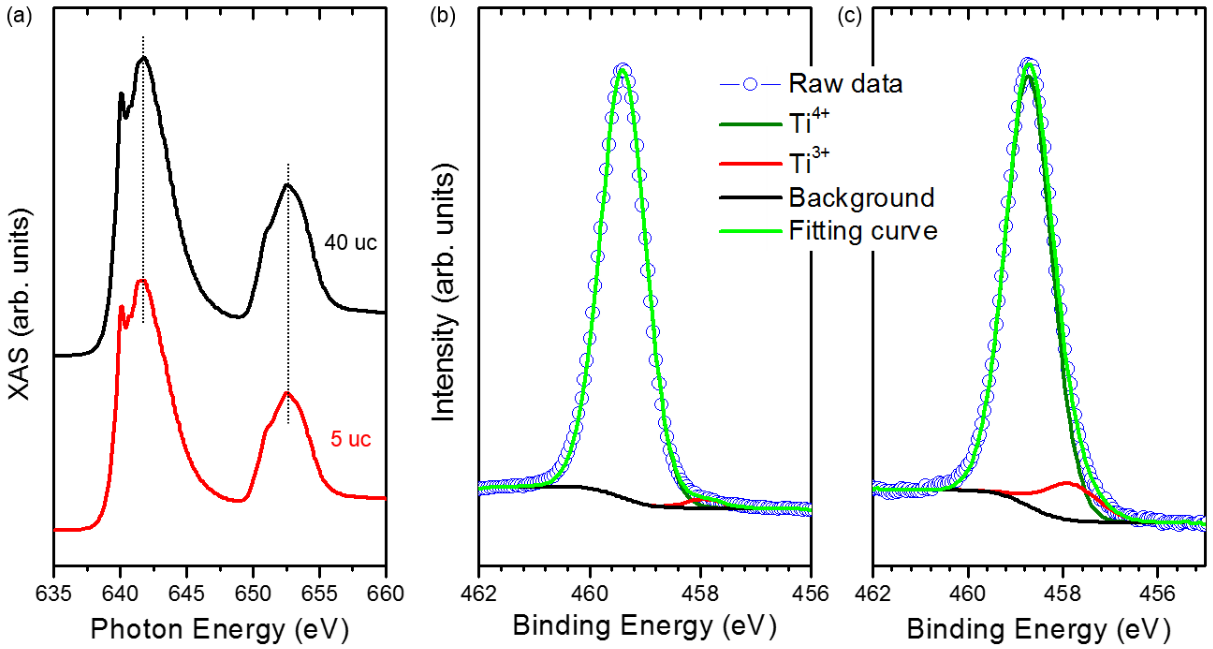

Figure S4. (a) Mn XAS spectra of 40 *uc* and 5 *uc* LBMO. Ti 2$p_{3/2}$ core-level spectra with peak fitting for (b) STO substrate and (c) 5 *uc* LBMO grown on STO substrate. There is a negligible intensity arising from Ti^{3+} oxidation state in the STO substrate. However, a clear shoulder related to Ti^{3+} state is observed in LBMO (5 *uc*)/STO, shown in Figure c.

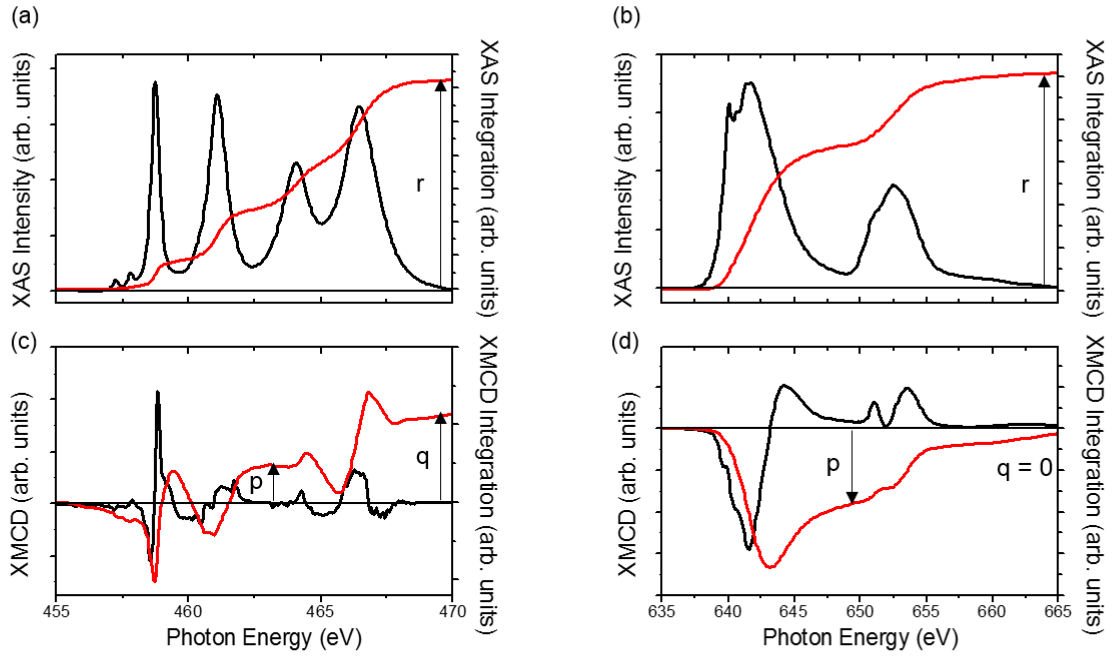

Figure S5. XAS (black) and integration (red) of XAS of Ti (a) and Mn (b) $L_{2,3}$ edges in the 5 *uc* LBMO film. XMCD (black) and integration (red) of XMCD of Ti (c) and Mn (d) $L_{2,3}$ edges in the 5 *uc* LBMO film.

To estimate the magnitude of the magnetic moment on the Ti sites and how the Ti moment is coupled with the Mn moment, the magneto-optical sum rules were applied to the XMCD data.[1] We should emphasize that the calculated magnetic moment value can be considered a lower bound. Previous work clearly demonstrates that sum rules applied to the light transition metal ions (like Ti) are known to seriously underestimate the moment due to the small spin orbital splitting of the $2p_{1/2}$ and $2p_{3/2}$ core levels.[2] The Eq.s of sum rules for spin and orbital moment are: [1]

$$m_{\text{spin}} = -\frac{6p-4q}{r}(10-n_{3d})$$

$$m_{\text{orb}} = -\frac{4}{3}\frac{q}{r}(10-n_{3d})$$

where n_{3d} is the 3*d* electronic density while *p*, *q*, and *r* are given by the integrations, as shown in Figure S5. The *q* value is positive in the integration of XMCD Ti (red in Figure S5c). However, the *q* value is very slightly negative for Mn (red in Fig. S5d) implying that the orbital moments of Ti and Mn align anti-parallel.

To calculate the spin magnetic moment, we have to know the number of electrons in the Ti and Mn 3*d* band. For the Mn, there are 4 electrons in 3*d* band assuming a Mn^{3+} configuration. However, for these mixed valence manganites there is also a small proportion of Mn^{4+} of the order of 10% as seen in the Mn XAS. For the Ti, the *in-situ* XPS Ti $2p_{3/2}$ core-level spectrum reveals that a small proportion of the Ti is in 3+ oxidation state (Figure S4c). Only Ti^{3+} contributes to the magnetic signal as there are no electrons in the Ti^{4+} 3*d* band. Assuming 1 electron in Ti^{3+} and 4 electrons in Mn^{3+} 3*d* band, based on the *p* and *q* values obtained from the integration of the XMCD, we obtain: $m_{\text{spin}} = 0.074\ \mu_B$/Ti and $m_{\text{spin}} = 2.59\mu_B$/Mn. The same sign in m_{spin} indicates that the orientation of Ti spin magnetic moment is same to the Mn magnetic moment. In other words, the Ti-O-Mn coupling is ferromagnetic at the LBMO/STO interface. Self-consistency is also seen be the anti-parallel alignment of the Ti spin and orbital which is to be expected from Hund's third rule (J=L-S) to shells that are less than half-filled, such as for Ti^{3+}.

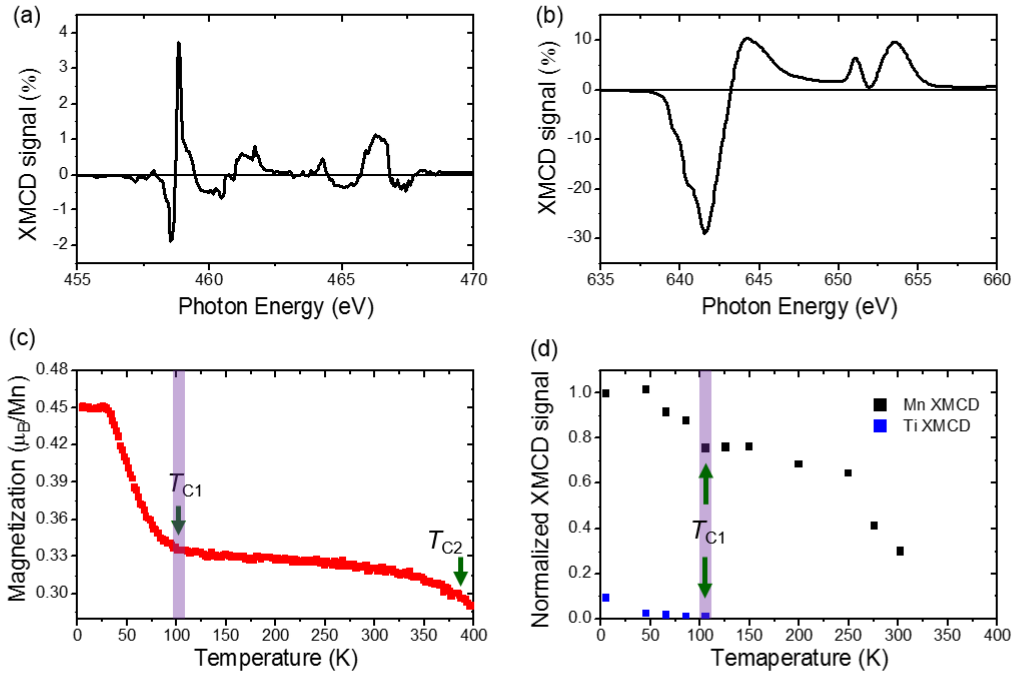

Figure S6. (a) Ti and (b) Mn XMCD signal of the 5 *uc* LBMO film measured at 2 K with a 4 T magnetic field. (c) Temperature dependent magnetization of the 5 *uc* LBMO film. (d) Temperature dependence of Ti (at ~ 466.35 eV) and Mn (at ~ 641.5 eV) XMCD signal for the 5 *uc* LBMO film.

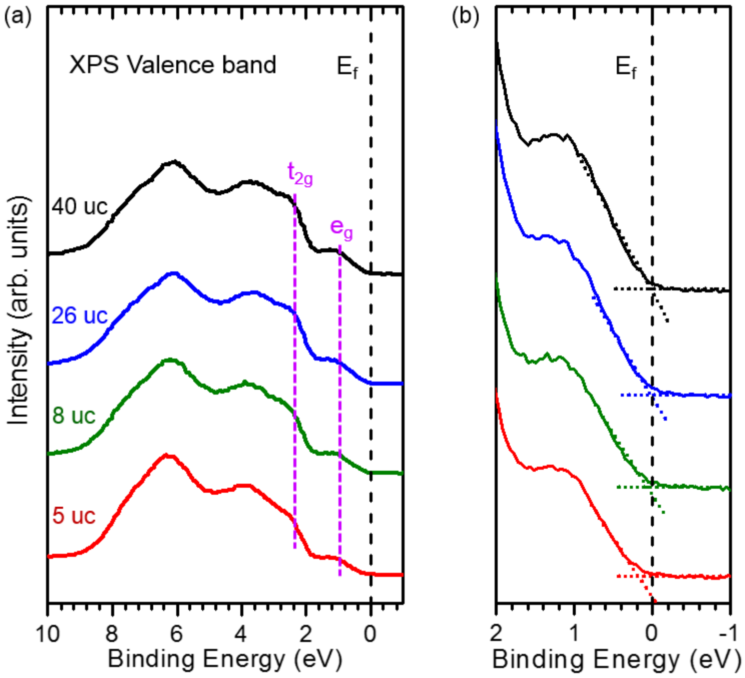

Figure S7. (a) *In-situ* XPS valence band spectra for LBMO with different thickness. (b) Enlarged view of XPS valence band spectra near the Fermi level (E_f). There is no intensity at E_f for the 5 *uc* LBMO, indicating the 5 *uc* LBMO films are insulating.

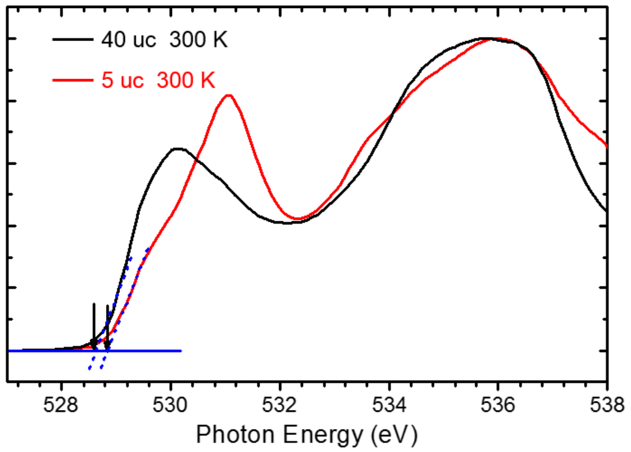

Figure S8. O K-edge XAS spectra for the 5 *uc* (red) and 40 *uc* (black) LBMO films. It clearly shows the pre-edge is shifted towards higher photon energy, suggesting the e_g bandwidth is reduced.

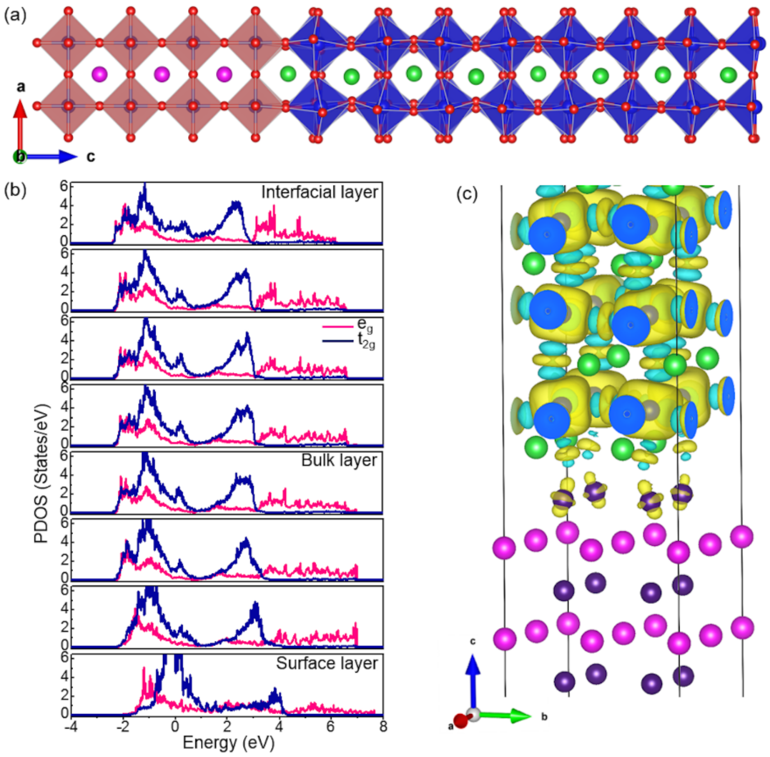

Figure S9. (a) The DFT relaxed structure of the LBMO-STO interface, looking down the [010] direction. The STO substrate consists of 2 × 2 × 4 unit cells and the LBMO film is modelled using 2 × 2 × 8 pseudo-cubic cells. The vacuum gap of 15 Å is not shown here. (b) Layer-by-layer projected density of states (PDOS) of Mn 3*d* electrons, separated into e_g and t_{2g} channels. A reduction of the e_g bandwidth is found at the interface. The Fermi level is set to be at 0 eV. (c) An isosurface plot of the spin density at the STO-LBMO interface. Positive and negative isosurface are labelled with yellow and blue colours. The shape of the isosurface at the interface Ti atoms resembles that of the $d_{3z^2-r^2}$ orbital. The magnetic interaction between Ti and Mn at the interface is ferromagnetic.

DFT calculations show that the LBMO-STO interface reduces the average OOP Mn-O-Mn rotation angle in the first two layers from the interface (Figure S9a). A similar effect also exists at the LBMO-vacuum interface. Partial Density of State (PDOS) analysis (Figure S9b) shows that the bandwidth of the Mn e_g orbitals is reduced at the LBMO-STO interface, which can explain the insulating property of the 5 *uc* films. In addition, the interface with STO substrate biases the occupation of e_g orbitals to the out-of-plane orientated $d_{3z^2-r^2}$ orbital, which is consistent with the XLD results. The Mulliken population analysis shows electrons are transferred to the Ti atoms at the interface. The latter becomes spin polarized and the spin density takes the shape of $d_{3z^2-r^2}$ (Figure S9c).

References

[1] C. T. Chen, Y. U. Idzerda, H.-J. Lin, N. V. Smith, G. Meigs, E. Chaban, G. H. Ho, E. Pellegrin, F. Sette, *Phys. Rev. Lett.* **1995**, *75*, 152.
[2] C. Piamonteze, P. Miedema, F. M. F de Groot, *Phys. Rev. B* **2009**, *80*, 184410.
